# Analysis of enzyme-responsive peptide surfaces by Raman spectroscopy†
†Electronic supplementary information (ESI) available: Detail information about materials and instrumentation, sample preparation, synthesis details and characterisation. See DOI: 10.1039/c5cc09189f



**DOI:** 10.1039/c5cc09189f

**Published:** 2016-03-08

**Authors:** Jugal Kishore Sahoo, Narayana M. S. Sirimuthu, Anne Canning, Mischa Zelzer, Duncan Graham, Rein V. Ulijn

**Affiliations:** a WestCHEM , Department of Pure & Applied Chemistry , Technology and Innovation Centre , University of Strathclyde , 99 George Street , Glasgow , G1 1RD , UK; b Centre of Molecular Nanometrology , WestCHEM , Department of Pure and Applied Chemistry , Technology and Innovation Centre , University of Strathclyde , 99 George Street , Glasgow , G1 1RD , UK . Email: Duncan.graham@strath.ac.uk; c Department of Chemistry , University of Sri Jayewardenepura , Sri Lanka; d University of Nottingham , School of Pharmacy , University Park , Boots Science Building , Nottingham , NG7 2RD , UK; e National Physical Laboratory , Hampton Rd , Teddington , Middlesex TW11 0LW , UK; f Advanced Science Research Center (ASRC) and Hunter College , City University of New York , NY 10031 , NY , USA . Email: Rein.Ulijn@asrc.cuny.edu

## Abstract

We report on the use of Raman spectroscopy as a tool to characterise model peptide functionalised surfaces. By taking advantage of Raman reporters built into the peptide sequence, the enzymatic hydrolysis of these peptides could be determined.

## Abstract 2

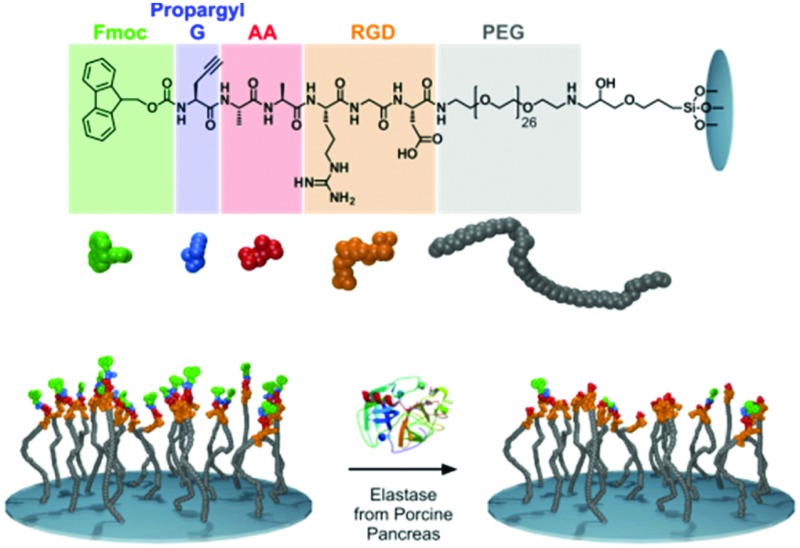
Detection of enzymatic hydrolysis of peptide surfaces by Raman spectroscopy.

The development of surfaces which are able to change their properties in response to biologically relevant cues is of interest in the context of dynamic biomaterials that adapt to their (biological) environment.^1–3^ In particular, stimuli-responsive surfaces,^4^ which have the ability to change their physical or chemical properties in response to an external stimulus, are gaining attention. The most common triggers include light,^5–7^ temperature,^8,9^ pH^10,11^ and electric field.^12–14^ In addition to these stimuli, enzymes provide an alternative, physiological trigger^1,15^ with the potential advantage of selectivity, inherent signal amplification, biocompatibility and ability to operate under constant, physiological conditions.

Although there are several characterisation techniques available to monitor and analyse the chemical composition of surfaces, such as X-ray photoelectron spectroscopy (XPS),^16–18^ solid state fluorescence spectroscopy^19^ and time-of-flight secondary ion mass spectroscopy (ToF-SIMS),^19^ there is a requirement for further analysis techniques to allow for readily accessible, non-invasive analysis of these surfaces. In the past, enzymatic hydrolysis at air/liquid interfaces has been monitored by Brewster angle microscopy and grazing incidence X-ray diffraction.^20–23^ Raman scattering is a vibrational spectroscopy technique, which can be used to identify chemical compounds or to monitor the change in the molecular composition of the matrix/substrate. Raman spectroscopy is non-invasive and routinely available technique which is easy to measure and analyse. In addition, Raman spectroscopy measurement is quick (seconds or milliseconds) and can be measured *in situ*. Here, we report the use of Raman spectroscopy to firstly analyse peptide functionalization in a semi-quantitative manner through detection of the Fmoc protecting group, followed by the analysis of enzymatic peptide hydrolysis using Raman reporters.

First, we produced simple peptide-functionalised surfaces, based on our previously reported methodology using a modification of fluorenyl methyloxycarbonyl (Fmoc) based Solid Phase Peptide Synthesis (SPPS) procedure.^19,26^ The amino acids were built-up in a stepwise manner directly onto amine terminated (PEG)_26_ chain functionalised on glass coverslips. Making use of the Raman vibrational modes of the Fmoc group we proposed to detect the presence/absence of the Fmoc-group (Fig. 1) as used previously to assess gelation and (chiral) molecular assembly of Fmoc-peptides. Previously, we demonstrated that surface functionalised Fmoc-peptides may be detected by solid-state fluorescence spectroscopy however this approach could not be used quantitatively.^19^ Quantitative measurement of components of mixtures of molecules can be made using Raman scattering and is compatible with both solids and liquids offering a complementary approach to fluorescence.

**Fig. 1:**
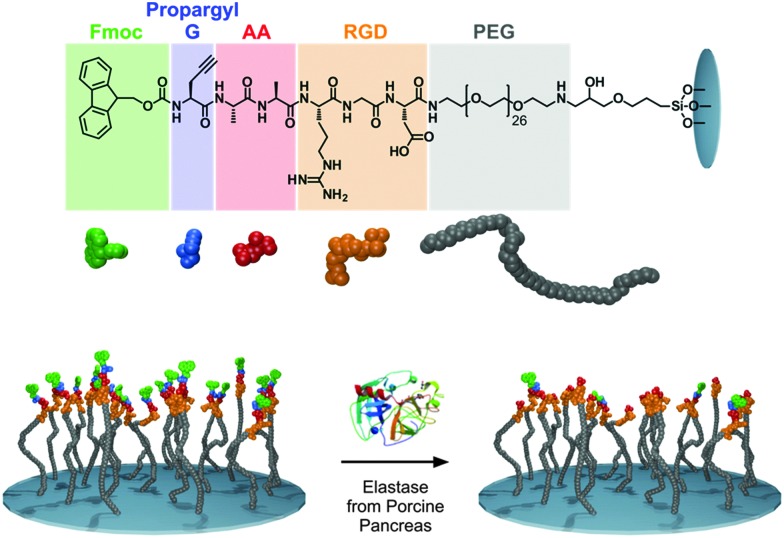
The chemical structure of the peptides synthesized (top), cartoon representation of the elastase responsive peptide surface, Fmoc-propargyl-GAARGD-(PEG)_26_ (bottom). After elastase exposure, the dialanine (AA) sequence is cleaved, removing Fmoc-propargyl GA and leaving ARGD-(PEG)_26_ on the surface.

For this study, we synthesized a peptide sequence on a surface (glass) which is a modification of our previously reported structures used in enzyme triggered cell attachment experiments^26^ and has the following features: (1) a terminal Fmoc propargyl glycine-group, which is used as the Raman marker, present at the N-terminus of the last amino acid of the sequence, (2) a dialanine (AA) sequence which may be cleaved by elastase from *porcine pancreas*,^26^ (3) a cell adhesive tripeptide sequence (RGD). The resulting full sequence on the surface is Fmoc-propargyl-GAARGD-(PEG)_26_ which was synthesised stepwise directly on the surface by SPPS as described in the Experimental section (ESI†). The surface used and the elastase catalysed removal of Fmoc-moieties is shown schematically in Fig. 1.

The synthesized surface Fmoc-propargyl-GAARGD-(PEG)_26_ and the surface obtained after exposure to elastase were characterised using ToF-SIMS and Raman spectroscopy. The presence and subsequent substantial reduction of signals attributed to the Fmoc-group after elastase treatment provides a suitable marker for the detection of the biocatalytic reaction on the surface. Fig. 2A displays the TOF-SIMS analysis of the elastase responsive surface. The fragment at *m*/*z* = 178 (C_10_H_14_^+^) was previously attributed to the Fmoc group.^19^ This peak was not detected on the control surfaces (Glass and (PEG)_26_). The observed intensity for the Fmoc marker subsequently decreases (70% ± 20%) after exposure to elastase, indicating cleavage of the sequence by the enzyme (Table S1, ESI†). The ToF-SIMS images for the Fmoc-marker and total ion intensity indicate that the surfaces are chemically homogeneous on the micrometer scale. Fragments associated with PEG (*e.g.* C_2_H_5_O^+^ at *m*/*z* = 45) increased significantly upon introduction of (PEG)_26_ on the surface and subsequently decreased in intensity when covered by the peptides (Fig. S2, ESI†). The ToF-SIMS data confirmed the presence and homogeneous surface distribution of Ala (A), Arg (R) and Gly (G) (Fig. S2, ESI†). Asp (D) or propargyl-glycine could not be unambiguously identified by ToF-SIMS as the fragments expected for these structures (*i.e.* C_3_H_3_^+^ at *m*/*z* = 39 and C_3_H_6_NO_2_^+^ at *m*/*z* = 88) are not specific and were also present on the control samples (glass and PEG).

**Fig. 2:**
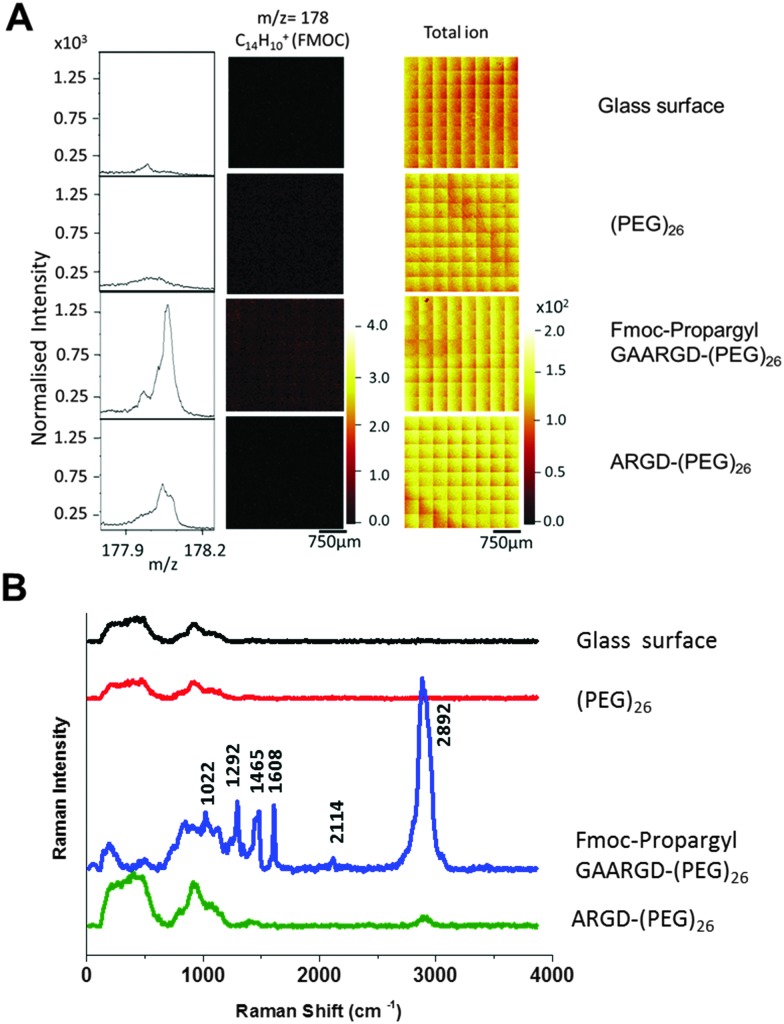
(A) ToF-SIMS spectra and images of the Fmoc related ion fragment at *m*/*z* = 178, (C_14_H_10_^+^) obtained from the samples before and after elastase treatment. The data was normalised to the total ion counts. (B) Raman spectra of enzyme responsive peptide surface (excitation wavelength-*λ*_ex_ = 532 nm, accumulation time 0.2 s).

Having verified the chemical composition of the surfaces, Raman spectroscopy was carried out on four different surfaces; (i) piranha cleaned glass surface, (ii) (PEG)_26_ functionalised glass surface, (iii) Fmoc-propargyl-GAARGD-(PEG)_26_ surface and (iv) Fmoc-propargyl-GAARGD-(PEG)_26_ surface after elastase exposure, *i.e.* ARGD-(PEG)_26_. (i) and (ii) are control surfaces before peptide build-up, (iii) and (iv) are before and after elastase exposure.

Fig. 2B displays the Raman spectra of the four different surfaces in the spectral region of 400–4000 cm^–1^. For the glass and (PEG)_26_ surfaces, we observe Raman signals at 500 and 922 cm^–1^ which are the background signals from glass probably due to silicates. For the Fmoc-propargyl-GAARGD-(PEG)_26_ surface, we observe Raman signals at 1022, 1292, 1465 and 1608 cm^–1^ which correlate to the known bands for the Fmoc group as reported earlier.^24^ The Raman band at 2114 cm^–1^ could be assigned to the C≡C stretching vibration, which was present in the propargyl group. The strong peak at 2892 cm^–1^ could be assigned to C–H stretching band (aromatic and aliphatic)^25^ from –Fmoc group and other amino acids. After elastase treatment, the peaks due to the Fmoc- and propargyl moieties reduce in intensity, which indicates successful cleavage of the AA bond by the enzyme, modifying the surface to ARGD-(PEG)_26_. The only Raman peaks we observe are due to glass and reduced C–H vibrations (due to removal of Fmoc-propargyl GA). From Raman spectroscopic data, it can be concluded that Fmoc is a better Raman reporter than the propargyl group for the peptide surfaces.

Having demonstrated that enzymatic hydrolysis can be followed with Raman spectroscopy, we moved on to assess whether the technique can be used to as a semi-quantitative method to verify chemical composition of peptide surfaces by using inherent Raman signals of amino acids. Thus, we synthesized a small set of peptide functionalised surfaces that use phenylalanine (Phe, F) as the Raman reporter. We synthesized two-component surfaces, composed of (F/A)-ARGD-(PEG)_26_ using four different ratios of F and A; 100 : 0, 75 : 25, 50 : 50, 25 : 75 (F : A). The surfaces were firstly characterised by ToF-SIMS analysis. Fig. 3B displays ToF-SIMS mass spectra and images of the C_8_H_10_N^+^ at *m*/*z* = 120 associated with Phe^27^ obtained from surfaces with different ratios of F/A. The Phe intensities show a uniform distribution over the surface and generally increase with increasing F content (Fig. 3D). The drop in intensity on the 100% F sample suggests that matrix effects may play a role in the ion yields on these samples. Matrix effects are commonly observed in ToF-SIMS analysis^28,29^ and we have observed similar effects on other types of peptide surfaces.

**Fig. 3:**
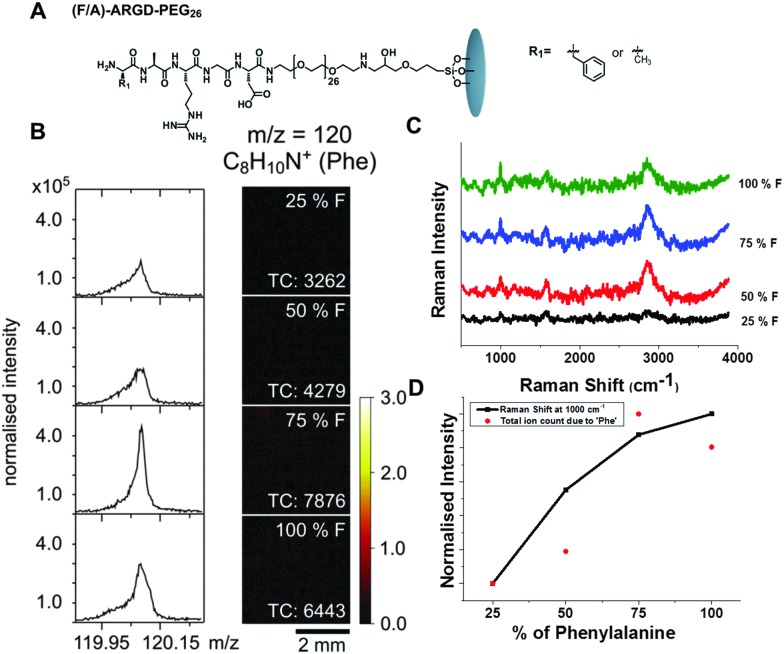
ToF-SIMS and Raman analysis of (F/A)-ARGD-(PEG)_26_ peptide surfaces with F/A ratios of 25/75, 50/50 and 75/25 and 100/0. (A) Chemical structure of the peptide surface. (B) Left hand side: Mass spectra of the fragment with *m*/*z* = 120 (C_8_H_10_N^+^, Phe). Right hand side: ToF-SIMS images of the surfaces. (TC = total counts). The data was normalised to the total ion counts. (C) Surface enhanced Raman spectra of the peptide surface with different ratios of F. (D) Intensities of the Phe fragment at *m*/*z* = 120 and the Phe related Raman line at 1000 cm^–1^. Both intensities were normalised to the highest values.

Fig. 3C shows the surface enhanced Raman spectroscopy (SERS) of the peptide surfaces. As Phe is a weaker Raman reporter compared to Fmoc-, there was a need to boost the Raman signal by using SERS^30^ (Fig. S3, ESI†) (SERS is typically performed by putting a drop of 20 nm gold colloid on the surfaces followed by drying^31^). Phe has characteristic Raman lines observed around 1000 cm^–1^ and 1605 cm^–1^.^32^ We also observe broad Raman line corresponding to C–H vibration from peptide surfaces at 2846–2855 cm^–1^. It can be observed that, with an increase in the concentration of Phe moieties on the surface, the intensity of the Raman lines due to phenylalanine increases (1000 cm^–1^). Fig. 3D displays an increase in Raman intensity at 1000 cm^–1^ with an increase in phenylalanine concentration on the surface. This clearly demonstrates the ability of Raman spectroscopy as a fingerprint technique to detect the molecular changes occurring on a functionalised surface.

In conclusion, we have successfully demonstrated, for the first time, that Raman spectroscopy can be used as characterisation technique to monitor and analyse peptide functionalised surfaces and detect enzymatic hydrolysis on the surface. Compared to other techniques, Raman spectroscopy is more easily accessible, inexpensive and offers non-invasive sample analysis. Notably, we were able to demonstrate that Raman spectroscopy can detect signals from thin peptide films and follow changes in the amino acid composition. This method will allow easy characterisation of these enzyme responsive surfaces and facilitate/promote wider use of peptide functionalised surfaces in different fields of biomaterials and bioengineering, and stem cell research^33,34^ including potential real-time analysis.

The authors declare no competing financial interest.

We thank the BBSRC for funding (BB/K007513/1) and the EPSRC for funding for AC (EP/F500491/1). The research leading to these results has received funding from the European Research Council under the European Union's Seventh Framework Programme (FP7/2007-2013)/ERC grant agreement no. 258775.
