# Supplementary material for: Analysis of enzyme-responsive peptide surfaces by Raman spectroscopy
Source: Chem Commun (Camb). 2016 Mar 8;52(25):4698–701. doi: 10.1039/c5cc09189f (PMC4819759; doi:10.1039/c5cc09189f)
Supplement: Supplementary file 1 [file CC-052-C5CC09189F-s001.pdf]

Electronic Supplementary Material (ESI) for ChemComm.
This journal is © The Royal Society of Chemistry 2016

# Supporting Information

## Analysis of enzyme-responsive peptide surfaces by Raman spectroscopy

Jugal Kishore Sahoo[a], Narayana M. S. Sirimuthu[b, f], Anne Canning[c], Mischa Zelzer[c, d], Duncan Graham[b*], Rein V. Ulijn[a, e*]

[a] WestCHEM, Department of Pure and Applied Chemistry, Technology and Innovation Centre, University of Strathclyde, 99 George Street, Glasgow, G1 1RD, UK.

[b] Centre of Molecular Nanometrology, WestCHEM, Department of Pure and Applied Chemistry, Technology and Innovation Centre, University of Strathclyde, 99 George Street, Glasgow, G1 1RD, UK.

[c] University of Nottingham, School of Pharmacy, University park, Boots Science Building, NG7 2RD, UK.

[d] National Physical Laboratory, Hampton Rd, Teddington, Middlesex TW11 0LW, UK

[e] Advanced Science Research Center (ASRC) and Hunter College, City University of New York, NY 10031, USA.

[f] Department of Chemistry, University of Sri Jayewardenepura, Sri Lanka.

**Contents**


1. **Materials and Methods**

   *Time of Flight Single Ion Mass Spectrometry (ToF-SIMS)*

   *Raman Spectroscopy*

   *Surface Enhanced Raman Spectroscopy*

2. **Experimental Section**

   *Solid Phase Peptide Synthesis*

   *Elastase Treatment*

3. **Figure S1: Chemical Scheme**

4. **Figure S2: ToF-SIMS of the Functionalised Surfaces**

5. **Figure S3: Comparison for Raman and SERS map for phenylalanine marker**

6. **Table S1: Intensities of mass fragments of modified surfaces.**

## Materials and Methods

**Time of Flight Secondary Ion Mass Spectrometry (ToF-SIMS)**. ToF-SIMS analysis was performed using a ToF-SIMS IV time-of-flight instrument (ION-TOF GmBH). An ION TOF, ToF SIMS IV instrument equipped with a Bi liquid metal ion gun (LMIG) was used to analyse the surfaces. A $Bi^{3+}$ primary ion source operating at 25 kV with a beam spot of 1-2 μm was used in high current bunched mode and directed at the surface at a 45 °C angle. A low energy electron flood gun was used to compensate for charging of the sample. Spectra were calibrated with m/z=1 ($H^+$), 15 ($CH_3^+$) 26 ($C_2H_5^+$) 43 ($C_3H_7^+$) 57 ($C_4H_9^+$). Spectra were normalised to total ion counts and analysed using Surface lab6 software. Numerical data reported was generated by the division of the images into quadrants using ROI editor on the duplicate samples to calculate average values.

**Raman Spectroscopy.** Raman analysis was carried out using WiTec alpha300 instrumentation, which facilitated the mapping of 30 x 30 μm areas. The false colour Raman map of intensity were obtained using a 532 nm excitation laser selected to match one of the Raman bands of the substrates. The parameters were set as following: $\lambda_{ex}$= 532 nm, 0.2 s, integration time, 100 x objectives. The intensity of the 1608 $cm^{-1}$ band was mapped resulting in a false colour image of the Raman enhancement over the analysis area. Three maps were taken from three separate, identically prepared samples. All maps from samples with the same time resembled one another to a high degree highlighting the reproducibility of the Raman response from the substrates. , Five Raman spectra were averaged to get a representative spectrum for each sample.

**Surface Enhanced Raman Spectroscopy.** To collect SERS spectra/maps from the surface, first a drop of Au colloid was added onto the surface of the sample, allowed to interact with the surface for 30 minutes and then pipetted out before SERS mapping. To collect a map 100X objective was used with 532 nm exciting laser at 0.2 s integration time. Au colloids were prepared according to the Lee and Meisel method[1]. Note that mapping was carried out while the area is still wet, this protocol was used to minimise the drying effect and forming unwanted hot-spots which give non-uniformity.

## Experimental Section

**Peptide Surface Preparation**. Glass coverslips (d= 13 mm, VWR) were cleaned by sonication in acetone, ethanol, methanol and milli-Q water ( respectively for 15 min each, followed by drying the coverslips in oven at 75 °C overnight, before cleaning in piranha solution). In a typical clean-up procedure, glass coverslips were immersed in piranha solution for 1 h before washing them in milli-Q water, followed by subsequent drying.

The dry glass coverslips were silanised by immersing them in (3-glycidyloxypropyl) trimethoxy silane for 1 h at 37 °C. After 1 h, coverslips were washed with acetone 3 times to remove unfunctionalised silane and then dried in an oven. $PEG_{26}$-diamine ($NH_2$-PEG-$NH_2$, Polypure, Norway) was melted onto the dry silanised coverslips at 75 °C and left to react in the oven for 48 h before washing the excess PEG away three times in milli-Q water. One amine group of the PEG-diamine is used to functionalise the silanised glass coverslips, leaving one free amine, which will be used subsequently for peptide synthesis.

For the peptide synthesis, the dry PEG amine functionalised glass coverslips were placed in a clean, dry glass petri dish. 20 mM Fmoc-amino acids solution was prepared in anhydrous Dimethyl formamide (DMF) containing 40 mM of ethyl (hydroxyimino) cyanoacetate (EHICA). The PEG-functionalised coverslips were immersed in Fmoc-amino acid solutions. To this solution, 2-equivalent of coupling agent (N, N'-diisopropyl carbodiimide; DIC) was added. The reaction proceeded at room temperature under gentle agitation (20 rpm) in an orbital shaker for 2h. The coverslips were washed in DMF, ethanol, methanol and DMF respectively for 5 min under gentle agitation and the same reaction was repeated and left to react overnight. In a similar washing procedure, the coverslips were washed in DMF, ethanol, methanol and DMF respectively for 5 min. The deprotection of the Fmoc-moieties was performed by immersing and gently shaking the coverslips with 20 % (v/v) piperidine solution in anhydrous DMF for 2h. After washing the surface in DMF, ethanol, methanol and DMF the next amino acid was functionalised using the same protocol.

After completion of the desired peptide sequence, the side groups of the amino acids were removed by immersing the coverslips in 90 % trifluoroacetic acid (TFA) in water for 4h. After completion of the deprotection, the coverslips were washed in a similar washing step as before (DMF, ethanol, methanol), followed by washing them in milli-Q water and blow dried in nitrogen followed by storing the sample in a polycarbonate desiccator in vacuum before further use.

**Elastase Treatment.** The terminal Fmoc-group was enzymatically cleaved by porcine pancreatic elastomer reconstituted in phosphate buffer saline (PBS). For elastase treatment, 2 ml of 0.1 mg/ml elastase was added to the coverslips in 24-cell plate and incubated at 37 °C for 48h followed by washing in ethanol, methanol and milli-Q water. The coverslips were dried in nitrogen and stored in a polycarbonate desiccator before further analysis.

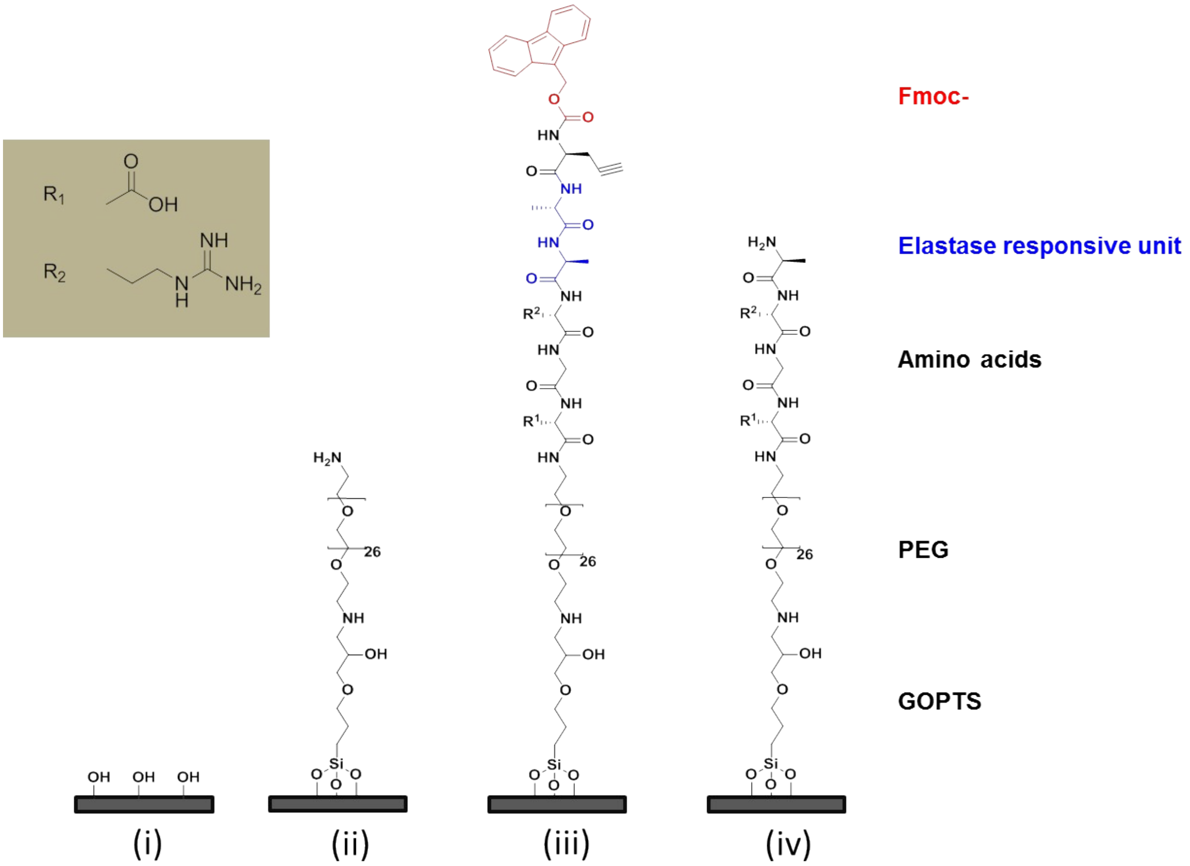


**Figure S1**: Chemical structure of the peptides synthesized via solid state peptide synthesis, (i) piranha cleaned glass coverslips; (ii) $(PEG)_{26}$ functionalised glass coverslips; (iii) Fmoc-Propargyl GAARGD-$(PEG)_{26}$ functionalised glass coverslips; (iv) peptide surface after elastase treatment.

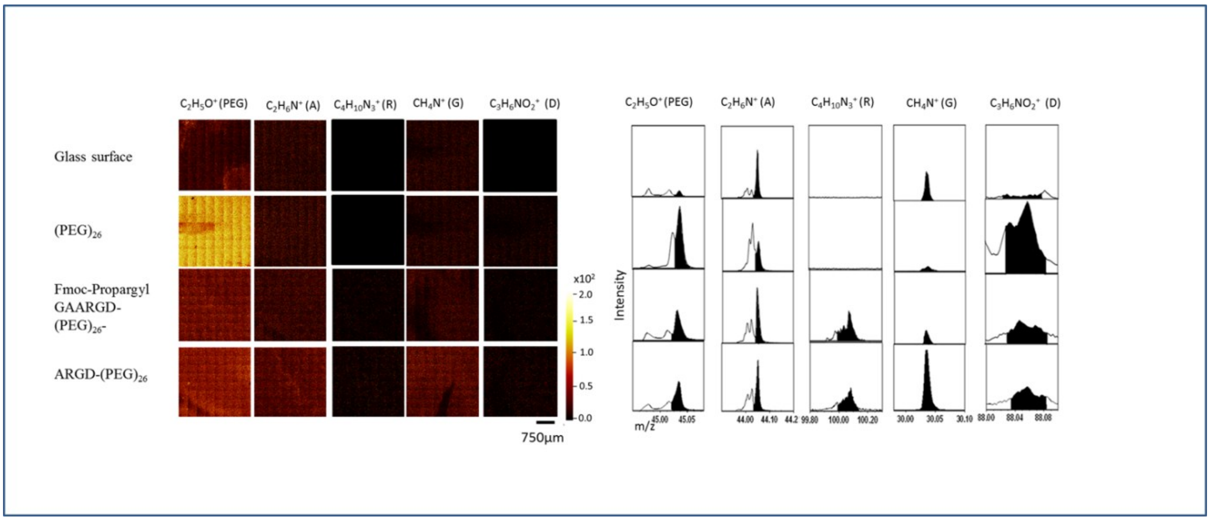


**Figure S2**: TOF SIMS analysis of the modified surfaces. Left: raster images of the surfaces, colour scale bar indicates ion counts. Right: Mass spectra of fragments associated with the amino acids, A: m/z= 44 ($C_2H_6N^+$), R: 100 ($C_4H_{10}N_3^+$), G: 30 ($CH_4N^+$), and PEG: 45 ($C_2H_5O^+$).

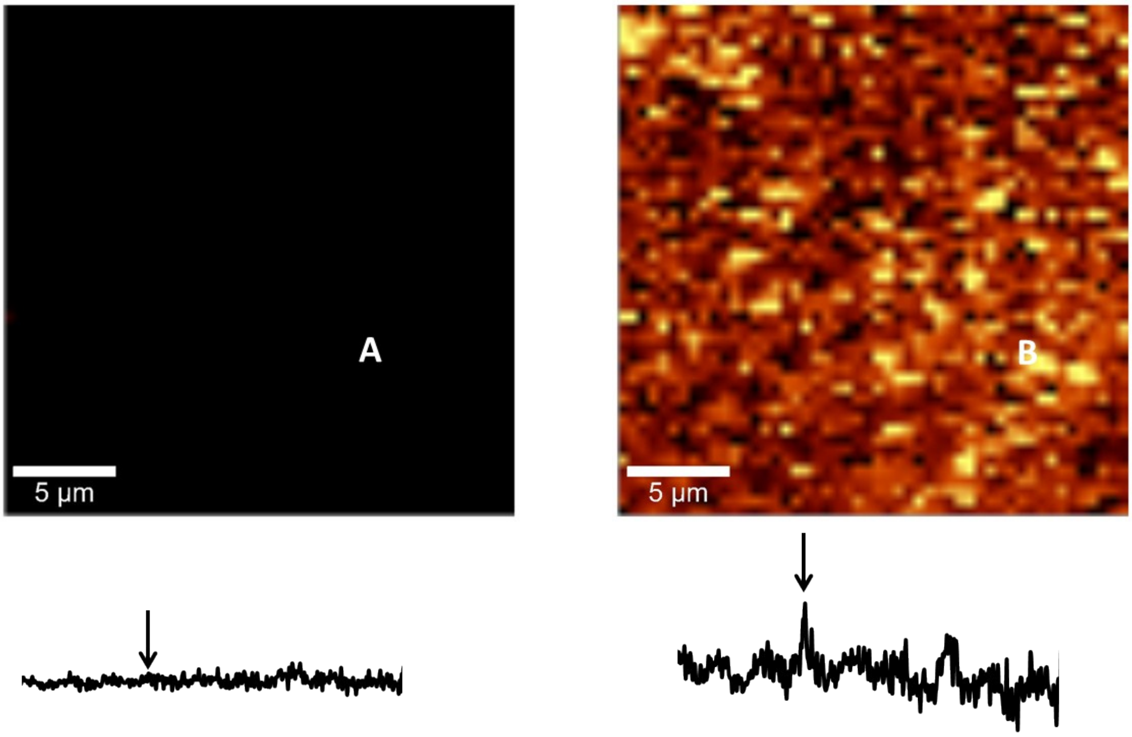


**Figure S3**: Raman map (left) obtained from the surface treated with phenylalanine, left bottom shows individual spectrum from point A in the map, indicating no peak at 1000 $cm^{-1}$ for –Phe. SERS map (right) obtained from the same surface after treatment with Au colloid, shows a distinct peak (1000 $cm^{-1}$).

**Table S1**: Intensities of mass fragments of modified surfaces normalised to total ion counts.

| | $CH_4N^+$ (G) | $C_2H_6N^+$ (A) | $C_2H_5O^+$ (PEG) | $C_3H_6NO_2^+$ (D) | $C_4H_{10}N_3^+$ (R) | $C_{14}H_{10}^+$ (Fmoc) |
|---|---|---|---|---|---|---|
| Glass | 3.69 ±0.40 | 3.21 ±0.25 | 3.87 ±1.33 | | | 0.16 ±0.01 |
| $(PEG)_{26}$ | 1.29 ±0.15 | 2.13 ±0.14 | 49.80 ±4.43 | | | 0.19 ±0.03 |
| Fmoc-Propargyl GAARGD-$(PEG)_{26}$ | 2.64 ±0.25 | 3.04 ±0.11 | 18.05 ±0.87 | 0.21 ±0.02 | 0.24 ±0.01 | 1.08 ±0.28 |
| ARGD-$(PEG)_{26}$ | 6.32 ±0.73 | 5.28 ±0.90 | 10.4 ±0.63 | 0.27 ±0.01 | 0.54 ±0.05 | 0.32 ±0.01 |

Reference:

1. P. C. Lee, D. Meisel, *J. Phys. Chem.*, 1982, **86** (17), 3391.
